# A network-based approach to dissect the cilia/centrosome complex interactome

**DOI:** 10.1186/2046-2530-4-S1-P87

**Published:** 2015-07-13

**Authors:** M Morleo, R Amato, L Giaquinto, D Di Bernardo, B Franco

**Affiliations:** 1 TIGEM, Pozzuoli, Italy; 2 Department of Medical Translational Sciences University of Naples "Federico II", Naples, Italy; 3 Department of Computer and Systems Engineering, University of Naples "Federico II", Naples, Italy

## Objective

We built a network of curated interactions between human proteins involved with centrioles, centrosomes, basal bodies and cilia to provide a global functional characterization of the Cilia/Centrosome Complex interactome (CCCI).

## Methods

Human ciliary genes were collected from available databases and interactions among genes were obtained from the STRING database (http://string-db.org). Network analyses were performed using Cytoscape and communities were extracted using the MCODE algorithm. The gene-wise network was explored by Gene Ontology (GO) analysis. The transcription factor (TF) analysis was performed using information obtained from "ConsSites" and "tfbsConsFactors" track of the UCSC Genome Browser.

## Results

We collected 3,502 ciliary genes and obtained the final CCCI, which consisted of 11,608 interactions among 1,695 selected genes. We identified 90 communities, groups of genes densely interconnected with each other and connected to few genes outside the group. We discovered communities specialized for delegating specific biological functions such as mRNA processing, protein translation, folding and degradation processes. In particular the "proteasome community" was enriched in ciliary components belonging to the SYSCILIA Gold Standard (SCGSv1) and in ciliopathy genes. We found 11 communities enriched in 30 TFs. The identified TFs are involved in developmental processes, cell cycle control, in the immune response and in muscle differentiation.

## Conclusions

CCCI is a publically available tool, which will allow to clarify the roles of previously unknown ciliary functions and to elucidate the molecular mechanisms underlying ciliary-associated phenotypes.

